# A case of hemobilia caused by pancreatic metastasis of renal cell carcinoma treated with a covered metallic stent

**DOI:** 10.1007/s12328-021-01532-1

**Published:** 2021-10-12

**Authors:** Masataka Yamawaki, Yuichi Takano, Jun Noda, Tetsushi Azami, Takahiro Kobayashi, Fumitaka Niiya, Naotaka Maruoka, Tatsuya Yamagami, Masatsugu Nagahama

**Affiliations:** Division of Gastroenterology, Department of Internal Medicine, Showa University Fujigaoka Hospital, 1-30 Fujigaoka, Aoba-ku, Yokohama-shi, Kanagawa, 227-8501 Japan

**Keywords:** Hemobilia, Renal cell carcinoma, Metallic stent

## Abstract

We present the case of an 86-year-old man who had undergone left nephrectomy for renal cell carcinoma (clear cell carcinoma) 22 years ago. He visited the emergency department complaining of right hypochondrial pain and fever. He was eventually diagnosed with acute cholangitis. Abdominal contrast-enhanced computed tomography showed multiple tumors in the pancreas. The tumor in the pancreatic head obstructed the distal bile duct. Endoscopic retrograde cholangiopancreatography detected bloody bile juice flowing from the papilla of Vater. Therefore, he was diagnosed with hemobilia. Cholangiography showed extrinsic compression of the distal bile duct; a 6 Fr endoscopic nasobiliary drainage tube was placed. Endoscopic ultrasound showed that the pancreas contained multiple well-defined hypoechoic masses. Endoscopic ultrasound-guided fine-needle aspiration was performed using a 22 G needle. Pathological examination revealed clear cell carcinoma, and the final diagnosis was pancreatic metastasis of renal cell carcinoma (RCC) causing hemobilia. A partially covered metallic stent was placed in the distal bile duct. Consequently, hemobilia and cholangitis were resolved.

## Introduction

Renal cell carcinoma (RCC) may recur even after a long period of time has passed from radical resection. However, it rarely results in pancreatic metastasis. Here we report a case of pancreatic metastasis of RCC that caused hemobilia 22 years after surgery; hemostasis was achieved by applying a covered-type self-expandable metallic stent (SEMS).

## Case report

The case was an 86-year-old man. Nephrectomy was performed for left renal tumor 22 years ago, and the patient was pathologically diagnosed with clear cell carcinoma. Five years after the surgery, follow-up was discontinued as there was no recurrence. He had an unremarkable family history. He consumed alcohol occasionally and had never smoked.

**Fig. 1:**
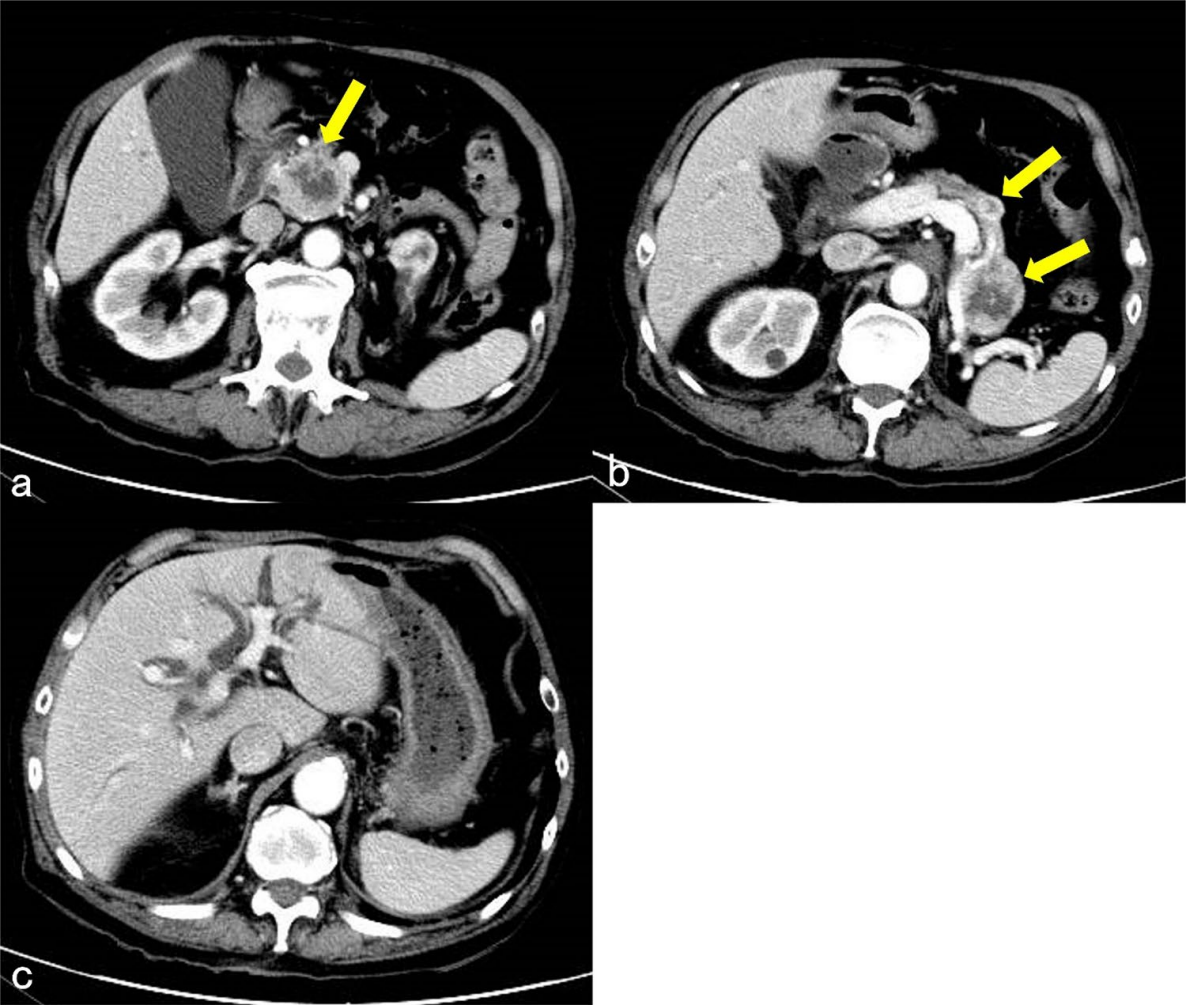
**a**, **b** Contrast-enhanced computed tomography showed multiple tumors in the pancreas (arrow). **c** The intrahepatic bile duct was dilated

**Fig. 2:**
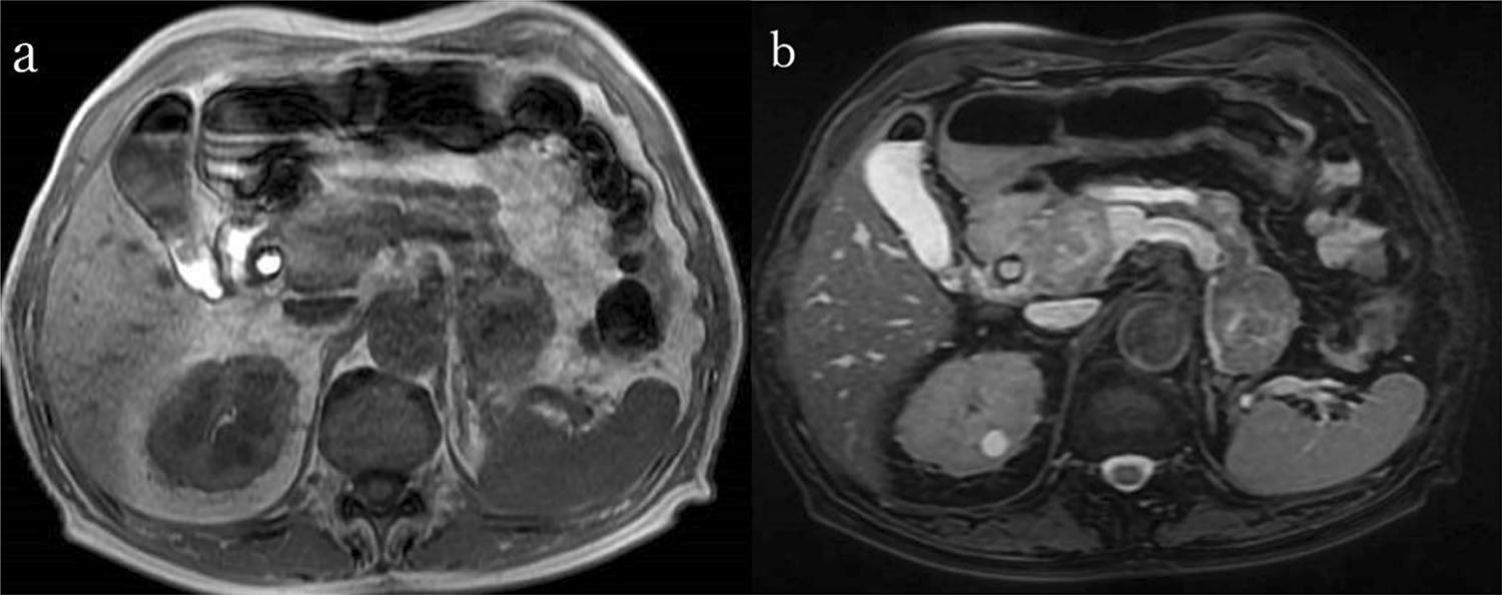
**a** Magnetic resonance imaging showed multiple tumors in the pancreas with heterogeneously low signal intensity on the T1-weighted image. Bile in the common bile duct and gallbladder neck has high intensity, which suggests hemobilia. **b** Pancreatic masses have rather low intensity on T2WI, suggesting intratumor bleeding. Area with high intensity in pancreas head mass is considered as tumor necrosis

**Fig. 3:**
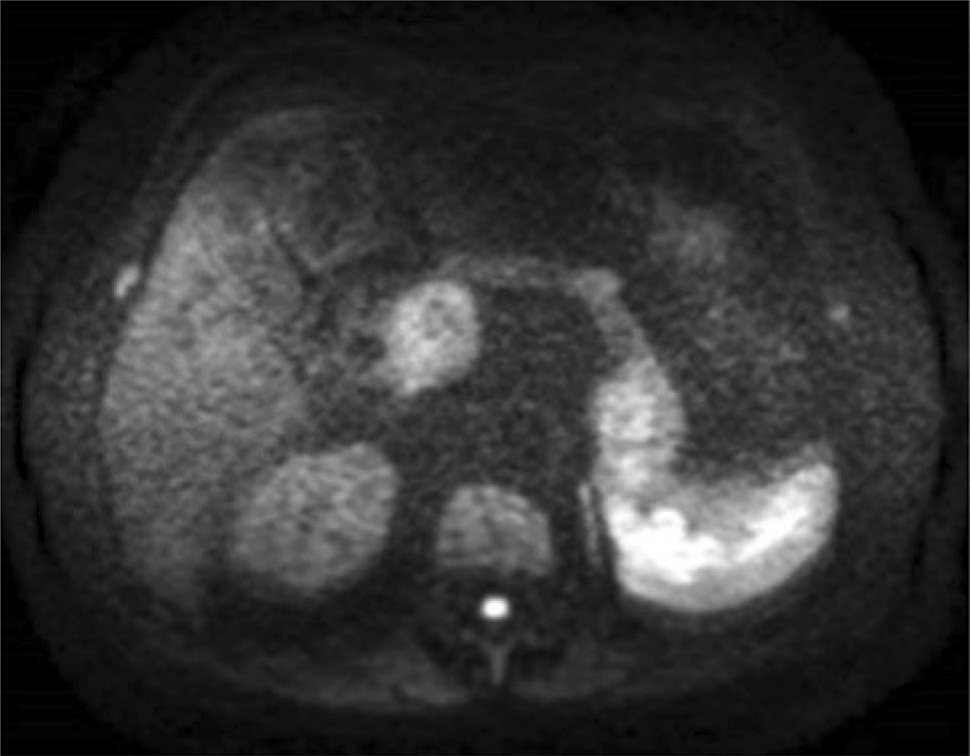
Diffusion-weighted magnetic resonance imaging (b-factor = 1000 s/mm2) showed multiple tumors in the pancreas with a high signal intensity. Apparent diffusion coefficient map revealed pancreatic tumors with low intensity

He visited the emergency department with complaints of right hypochondrial pain and fever. His vital signs at admission were as follows: body temperature, 38.5 °C; blood pressure, 94/52 mmHg; pulse rate, 114 beats/min; saturation of peripheral oxygen, 96% on room air; and respiratory rate, 18 cycles/min. Physical findings revealed conjunctival icterus and right hypochondrial tenderness. The results of a blood test showed decreased white blood cell count (2430 cells/μL); however, the C-reactive protein level was normal (0.16 mg/dL). In addition, the levels of total bilirubin (2.0 mg/dL), direct bilirubin (1.2 mg/dL), aspartate aminotransferase (736 U/L), alanine transaminase (350 U/L), alanine transaminase (478 U/L), and gamma-glutamyl transpeptidase (281 U/L) increased. Meanwhile, the results for the tumor markers were within normal ranges (cancer antigen 19–9, < 37 U/mL; carcinoembryonic antigen, 1.3 ng/dL; and Dupan-2, 36 U/mL) (Table 1). Abdominal contrast-enhanced computed tomography showed multiple tumors in the pancreas. In particular, a tumor in the pancreatic head compressed the bile duct, and the intrahepatic/extrahepatic bile duct was dilated (Fig. 1). Magnetic resonance imaging showed multiple tumors in the pancreas with heterogeneously low signal intensity on the T1-weighted image. Bile in the common bile duct and gallbladder neck has high intensity, which suggests hemobilia (Fig. 2a). Pancreatic masses have rather low intensity on T2WI, suggesting intratumor bleeding. Area with high intensity in pancreas head mass is considered as tumor necrosis (Fig. 2b). Diffusion-weighted magnetic resonance imaging (b-factor = 1000 s/mm2) showed multiple tumors in the pancreas with a high signal intensity (Fig. 3). Apparent diffusion coefficient map revealed pancreatic tumors with low intensity.

**Table 1:** Laboratory findings

Complete blood count		Chemistry			
White blood cells	2430/μL	Total protein	6.6 g/dL	Sodium	142 mEq/L
Red blood cells	399 × 10^4^/μL	Albumin	4.2 g/dL	Potassium	4.8 mEq/L
Hemoglobin	11.9 g/dL	Total bilirubin	2.0 mg/dL	Chloride	112 mEq/L
Platelets	11.1 × 10^4^/μL	Direct bilirubin	1.2 mg/dL	CA19-9	< 37U/mL
		AST	736 IU/L	CEA	1.3 ng/dL
		ALT	350 IU/L	Dupan-2	36U/mL
Coagulation		ALP	478U/L	HBs-Ag	(–)
PT-INR	1.05	γGTP	281U/L	HCV-Ab	(–)
APTT	22.7 s	LD	675U/L		
		Amylase	255U/L		
		BUN	30.3 mg/dL		
		Creatinine	1.10 mg/dL		
		Uric acid	6.1 mg/dL		
		CRP	0.16 mg/dL		
		Procalcitonin	1.05 ng/mL		

*PT-INR* prothrombin time-international normalized ration, *APTT *activated partial thromboplastin time, *AST *aspartate aminotransferase, *ALT *alanine, *ALP* alkaline phosphatase aminotransferase, *γGTP* gamma-glutamyl transpeptidase, *LD* lactate dehydrogenase, *BUN* Blood urea nitrogen, *CRP* c-reactive protein, *CA19-9* carbohydrate antigen 19–9, *CEA* carcinoembryonic antigen, *Dupan-2 *duke pancreatic monoclonal antigen type2, *HBsAg* hepatitis B surface antigen, *HCVAb* hepatitis C antibody

**Fig. 4:**
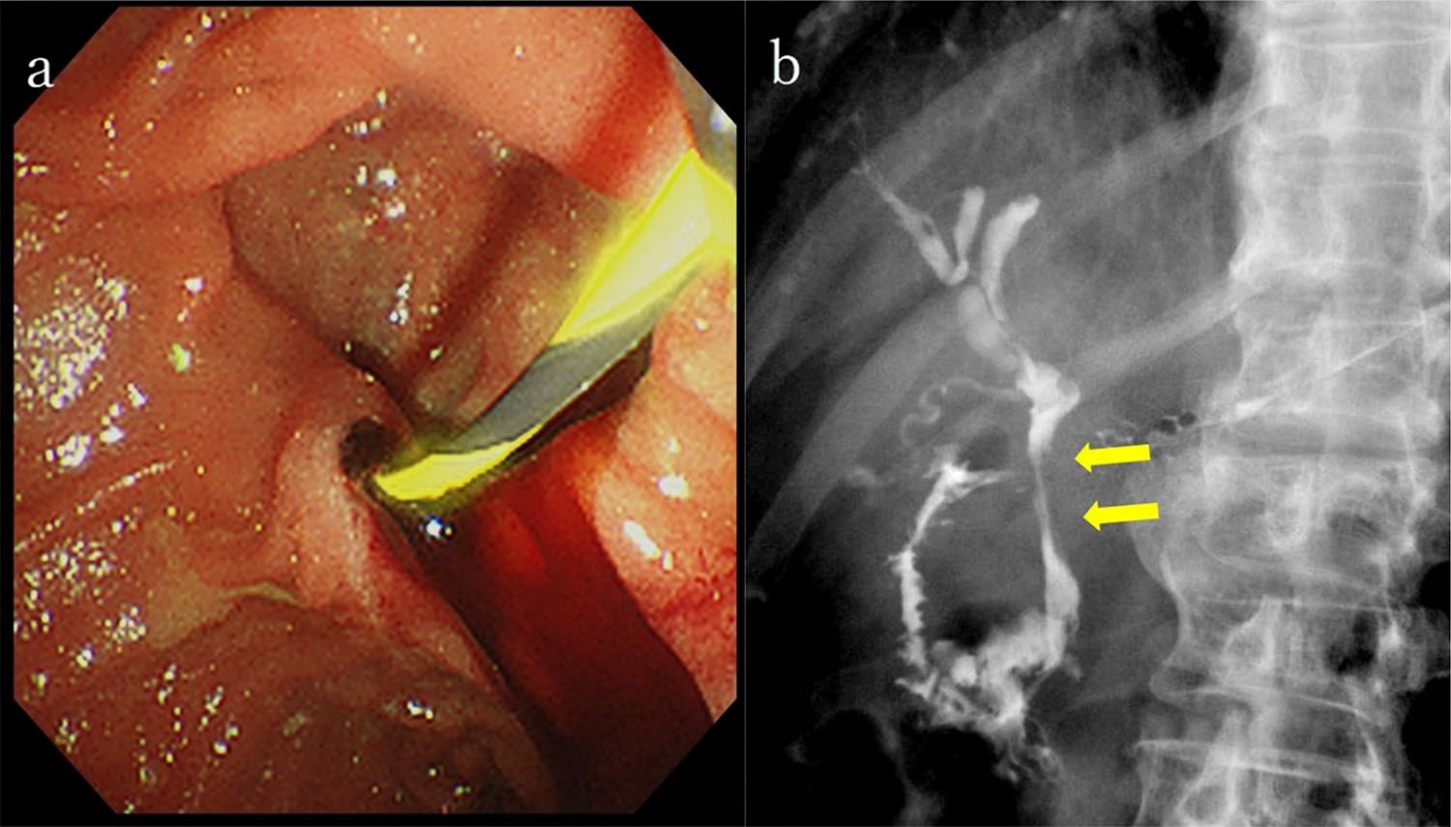
Endoscopic retrograde cholangiopancreatography findings. **a** Hemobilia was observed after biliary cannulation. **b** Cholangiography via the endoscopic naso-biliary drainage revealed that the distal bile duct was extrinsically compressed by the tumor (yellow arrow)

**Fig. 5:**
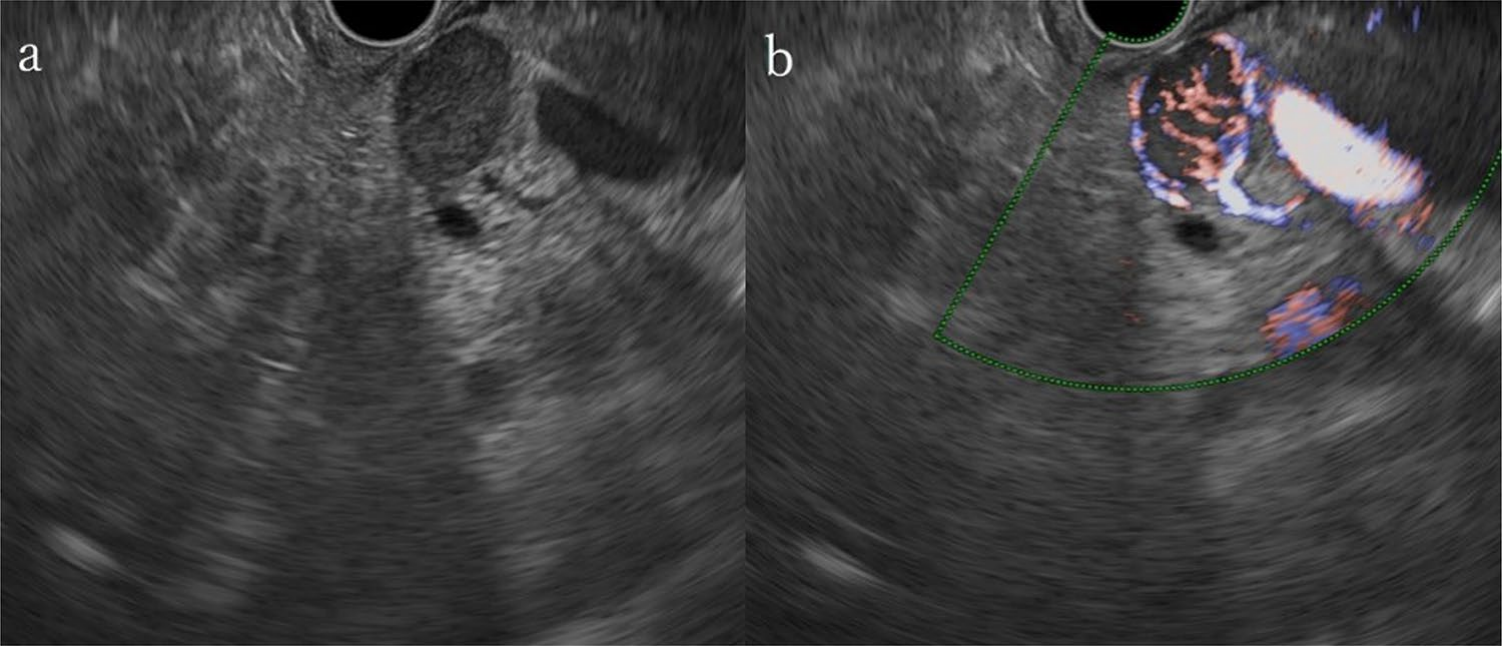
Endoscopic ultrasound showed **a** multiple well-defined hypoechoic masses in the pancreas, and **b** color Doppler ultrasound showed abundant blood flow inside the masses

**Fig. 6:**
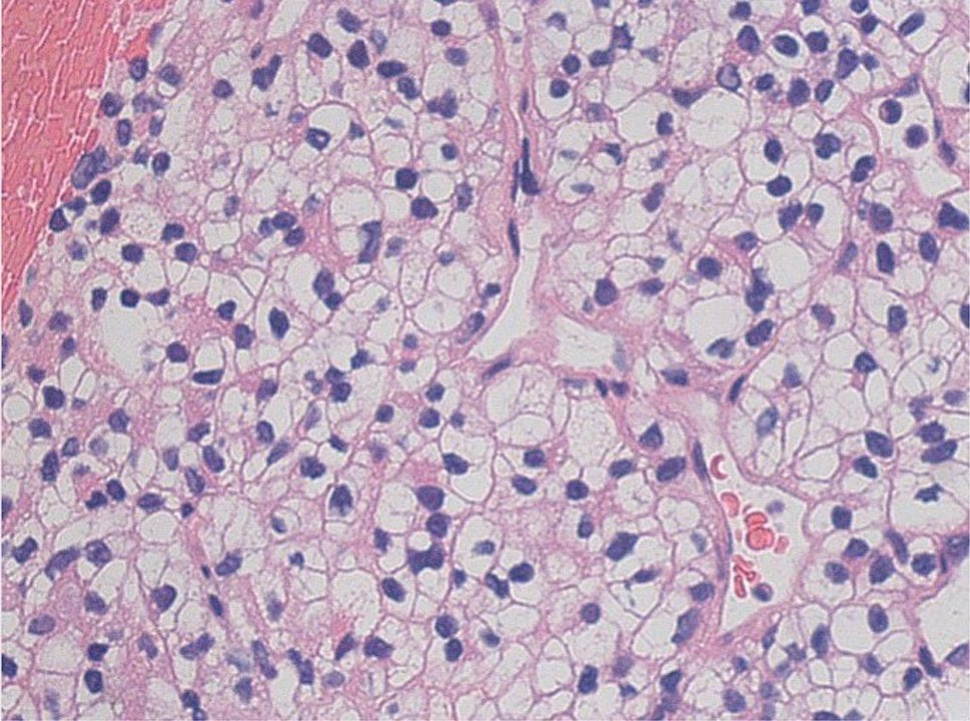
Pathological findings of the specimen obtained by endoscopic ultrasound-guided fine needle aspiration. Hematoxylin–eosin staining revealed clear cell carcinoma with a clear cytoplasm and rich sinusoidal vasculature

The patient was diagnosed with acute cholangitis (Tokyo Guidelines 2018, Grade II of severity criteria [1]). Subsequently, endoscopic retrograde cholangiopancreatography (ERCP) was performed, revealing the flow of bloody bile juice from the papilla of Vater; therefore, the patient was diagnosed with hemobilia. A 6 Fr endoscopic nasobiliary drainage (ENBD) tube was placed. Cholangiography via the endoscopic naso-biliary drainage revealed that the distal bile duct was extrinsically compressed by the tumor (Fig. 4). Acute cholangitis improved after ENBD placement, but hemibilia persisted. Endoscopic ultrasound (EUS) detected multiple well-defined hypoechoic masses in the pancreas, and Doppler ultrasound showed abundant blood flow inside the mass (Fig. 5a, b). A tumor in the pancreatic tail underwent EUS-guided fine-needle aspiration (EUS-FNA) using a 22 G needle (Expect™ SlimLine, Boston Scientific Japan, Tokyo, Japan). Pathological examination revealed round-shaped tumor cells with clear cytoplasm, indicating clear cell carcinoma (Fig. 5c). Surgical and pathological reports of surgical resection of RCC 22 years ago were not available, but based on the clinical images and pathological findings of EUS-FNA, the patient was diagnosed with multiple pancreatic metastases from RCC. Another ERCP was performed, and a partially covered SEMS with 10 mm diameter and 80 mm length (WallFlex™ Biliary RX Stent, Boston Scientific Japan, Tokyo, Japan) was placed after small-incision endoscopic sphincterotomy (Fig. 6). Postoperatively, hemobilia and acute cholangitis were resolved. One month after the SEMS placement, the hemoglobin level increased to 12.5 g/dL.

**Fig. 7:**
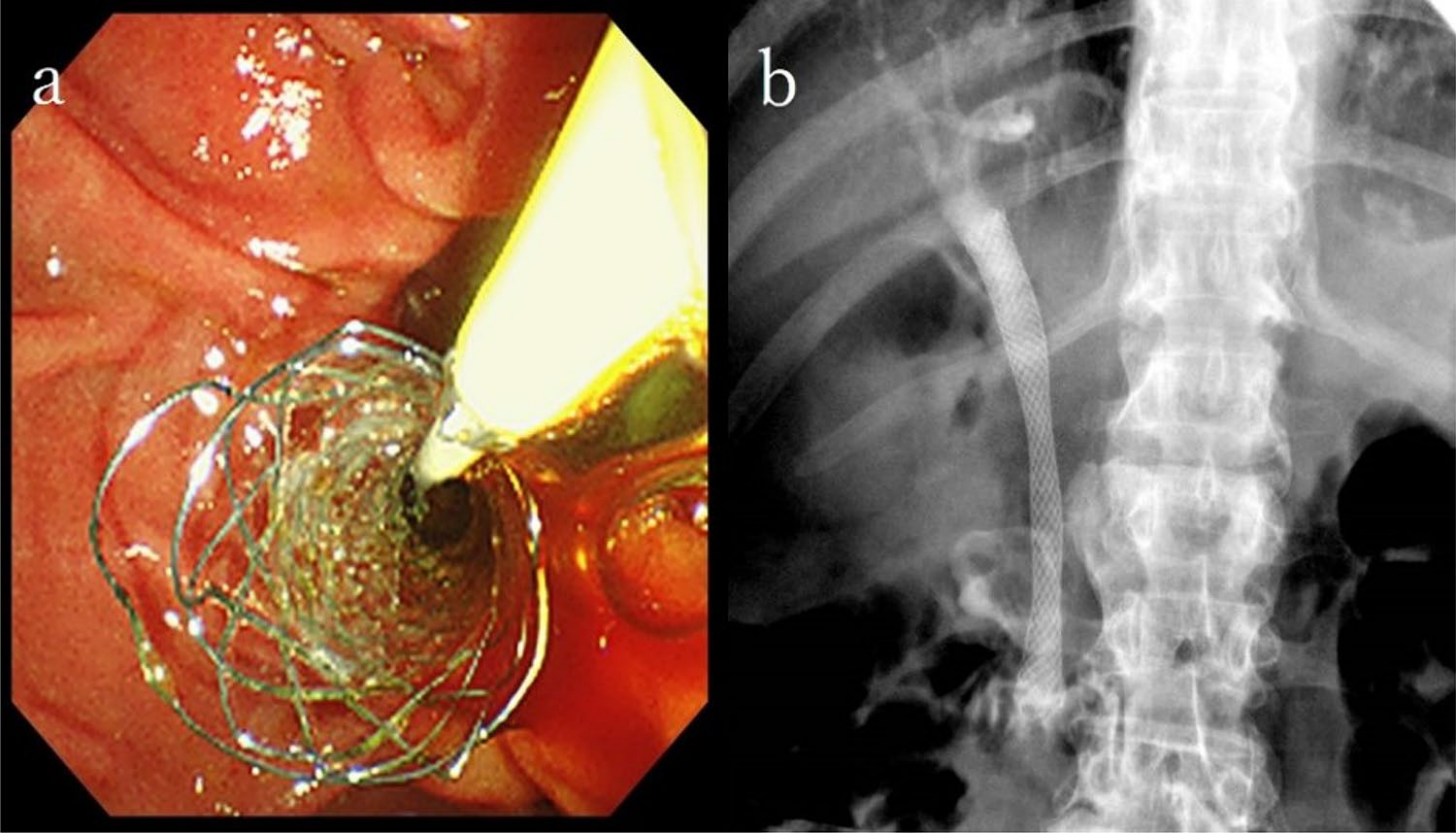
**a** After diagnosis, endoscopic retrograde cholangiopancreatography was performed, and a partially covered self-expandable metallic stent was placed. **b** Cholangiography revealed that the stenosis of the middle bile duct improved

Considering that the patient and his family refused chemotherapy, palliative treatment was continued. Two years have passed, since the stent was placed, no SEMS migration has occurred, and no hemobilia nor acute cholangitis has been observed, Fig. 7.

## Discussion

RCC mostly metastasizes in the lung (45.2%), bone (29.5%), lymph nodes (21.8%), liver (20.3%), adrenal gland (8.9%), and brain (8.1%) and rarely in the pancreas [2]. In previous reports, the rate of pancreatic metastasis was approximately 3% [3]; 2–5% of all pancreatic malignancies were metastatic tumors [4]. Meanwhile, among the surgically resected metastatic pancreatic tumors, those with primary RCC are the most common, accounting for 58.6–70% [5–7].

In cases of pancreatic metastasis of RCC, the mean period from nephrectomy to pancreatic metastasis is 6.9–14.6 years [8–13]. In some cases, pancreatic metastasis was even confirmed after 32.7 years of remission [12]. Therefore, despite reaching 22 years postoperatively, as in the present case, pancreatic metastasis should be considered in patients with a history of RCC.

Hemobilia is a rare condition that was first reported by Sandblom in 1948 [14]. Iatrogenicity is the most well-known cause of biliary bleeding (65%), whereas neoplasm is relatively rare (7%) [15]. Of the malignant neoplasms causing hemobilia, hepatocellular carcinoma, cholangiocarcinoma, gallbladder cancer, and pancreatic cancer are the most common, accounting for 50%, 35.7%, 7.1%, and 7.1%, respectively, whereas RCC causing hemobilia is rare [16].

A literature search for “hemobilia, renal cell carcinoma” in PubMed revealed only one case of hemobilia caused by RCC [17]. Herein, hemobilia occurred because of bleeding from a metastatic gallbladder tumor from RCC. RCC is a hypervascular tumor that can cause tumor bleeding. It often grows by expansion rather than infiltration. In the present case, a metastatic tumor in the pancreatic head compressed the distal bile duct severely, causing a minor perforation of the bile duct wall and exposing the tumor into the bile duct. Although direct cholangioscopy could be useful for definitive diagnosis, securing a visual field may be difficult when hemobilia persists; thus, the policy was to prioritize hemostasis and biliary drainage. Hemostasis cannot be achieved using a plastic stent; hence, we used a partially covered SEMS, which successfully stopped the bleeding.

There are two types of covered SEMS: fully covered SEMS and partially covered SEMS. The present case showed a “compressive” biliary stenosis, which may be more loosened than the “invasive” stenosis, such as that in pancreatic cancer. Therefore, a partially covered SEMS, with uncovered parts at both ends, was used to anchor the stent. Two years have passed since the procedure, but no stent migration has been observed.

We searched PubMed for cases in which hemobilia was managed with a metallic stent from 2000 to 2020 with the keyword "haemobilia, metallic stent". As a result, nine cases were collected, including our case (Table 2). The average age is 67 years, male 7 cases, female 2 cases, primary disease is cholangiocarcinoma 2 cases, portal biliopathy 2 cases, liver metastasis of rectal cancer1 case, hepatocellular carcinoma 1 case, pancreatic cancer 1 case, gallbladder cancer 1 case, and pancreatic metastasis of renal cell carcinoma 1 case. The stent type was fully covered in 7 cases, partially covered in 2 cases, and the stent location was distal bile duct in 5 cases, hilar in 3 cases, and hilar to distal in 1 case. Hemostasis was successful in all cases, and no complications were found. Metallic stents for hemobilia seems to be an effective treatment. In the covered type, the stent presses on the surrounding tissue, which can perform compression hemostasis.

**Table 2:** Cases of hemobilia managed with metallic stent

Author	Year	Reference	age, sex	Cause of hemobilia	Stent	Stent location	Hemostasis	Complication
Rerknimitr et al	2007	[18]	66, male	Liver metastasis of rectal cancer	Partially covered	Hilar	Success	none
Layec et al	2009	[19]	74, female	Portal biliopathy	Fully covered	Distal	Success	none
Bagla et al	2012	[20]	65, male	Cholangiocarcinoma	Fully covered	Hilar to distal	Success	none
Kawaguchi et al	2012	[21]	63, male	Hepatocellular carcinoma	Fully covered	Hilar	Success	none
Goenka et al	2014	[22]	22, male	Portal bilopathy	Fully covered	Distal	Success	none
Barresi et al	2015	[23]	67, male	Panceratic head cancer*	Fully covered	Distal	Success	none
Zhang et al	2018	[24]	90, female	Gallbladder cancer	Fully covered	Distal	Success	none
Tien et al	2020	[25]	70, male	Cholangiocarcinoma	Fully covered	Hilar	Success	none
Our case	2021	–	86, male	Pancreatic metastasis of renal cell carcinoma	Partially covered	Distal	Success	none

^*^Hemobilia was seen after endoscopic ultrasound-guided fine needle aspiration

## Conclusion

This report presents a case of hemobilia caused by pancreatic metastasis of RCC. Pancreatic metastases should be considered in patients with a RCC history, even long after surgery. EUS-FNA effectively confirmed the histological diagnosis, and covered-SEMS compression was effective for hemobilia caused by pancreatic head metastatic tumor.
